# Impact of Three Rounds of Mass Drug Administration on Lymphatic Filariasis in Areas Previously Treated for Onchocerciasis in Sierra Leone

**DOI:** 10.1371/journal.pntd.0002273

**Published:** 2013-06-13

**Authors:** Joseph B. Koroma, Santigie Sesay, Mustapha Sonnie, Mary H. Hodges, Foday Sahr, Yaobi Zhang, Moses J. Bockarie

**Affiliations:** 1 National Neglected Tropical Diseases Control Programme, Ministry of Health and Sanitation, Freetown, Sierra Leone; 2 African Programme for Onchocerciasis Control, Ouagadougou, Burkina Faso; 3 Centre for Neglected Tropical Diseases, Liverpool School of Tropical Medicine, Liverpool, United Kingdom; 4 Helen Keller International, Freetown, Sierra Leone; 5 College of Medicine and Allied Health Sciences, University of Sierra Leone, Freetown, Sierra Leone; 6 Helen Keller International, Regional Office for Africa, Dakar, Senegal; Liverpool Associates in Tropical Health, United Kingdom

## Abstract

**Background:**

1974–2005 studies across Sierra Leone showed onchocerciasis endemicity in 12 of 14 health districts (HDs) and baseline studies 2005–2008 showed lymphatic filariasis (LF) endemicity in all 14 HDs. Three integrated annual mass drug administration (MDA) were conducted in the 12 co-endemic districts 2008–2010 with good geographic, programme and drug coverage. Midterm assessment was conducted 2011 to determine impact of these MDAs on LF in these districts.

**Methodology/Principal Findings:**

The mf prevalence and intensity in the 12 districts were determined using the thick blood film method and results compared with baseline data from 2007–2008. Overall mf prevalence fell from 2.6% (95% CI: 2.3%–3.0%) to 0.3% (95% CI: 0.19%–0.47%), a decrease of 88.5% (p = 0.000); prevalence was 0.0% (100.0% decrease) in four districts: Bo, Moyamba, Kenema and Kono (p = 0.001, 0.025, 0.085 and 0.000 respectively); and seven districts had reductions in mf prevalence of between 70.0% and 95.0% (p = 0.000, 0.060, 0.001, 0.014, 0.000, 0.000 and 0.002 for Bombali, Bonthe, Kailahun, Kambia, Koinadugu, Port Loko and Tonkolili districts respectively). Pujehun had baseline mf prevalence of 0.0%, which was maintained. Only Bombali still had an mf prevalence ≥1.0% (1.58%, 95% CI: 0.80%–3.09%)), and this is the district that had the highest baseline mf prevalence: 6.9% (95% CI: 5.3%–8.8%). Overall arithmetic mean mf density after three MDAs was 17.59 mf/ml (95% CI: 15.64 mf/ml–19.55 mf/ml) among mf positive individuals (65.4% decrease from baseline of 50.9 mf/ml (95% CI: 40.25 mf/ml–61.62 mf/ml; p = 0.001) and 0.05 mf/ml (95% CI: 0.03 mf/ml–0.08 mf/ml) for the entire population examined (96.2% decrease from baseline of 1.32 mf/ml (95% CI: 1.00 mf/ml–1.65 mf/ml; p = 0.000)).

**Conclusions/Significance:**

The results show that mf prevalence decreased to <1.0% in all but one of the 12 districts after three MDAs. Overall mf density reduced by 65.0% among mf-positive individuals, and 95.8% for the entire population.

## Author Summary

Onchocerciasis studies across Sierra Leone between 1974 and 2005 showed that 12 of the 14 health districts (HDs) are endemic for onchocerciasis. Baseline lymphatic filariasis (LF) studies 2005–2008 showed that all 14 HDs of Sierra Leone are LF endemic. Three annual rounds of integrated mass drug administration (MDA) with ivermectin and albendazole 2008–2010 were conducted in the 12 HDs that are co-endemic for onchocerciasis and LF with good geographic, epidemiological drug (or programme) and drug coverage. A midterm evaluation study of mf prevalence and density was conducted in the 12 HDs in 2011. The hypothesis proposed for this study is that areas previously exposed to ivermectin treatment for onchocerciasis control may require less rounds of annual MDA to eliminate LF (i.e. reduce microfilaremia (mf) prevalence to <1%). Results of the midterm evaluation study showed very significant and rapid reduction of mf prevalence and density with 11 out of the 12 districts having mf prevalence <1%. Relatively low LF baseline prevalence and effective integrated MDA for onchocerciasis and LF have led to rapid reduction in LF prevalence.

## Introduction

Lymphatic filariasis (LF) and onchocerciasis are two of the major neglected tropical diseases (NTDs), presently targeted for elimination using the World Health Organization (WHO) recommended strategy of preventive chemotherapy and transmission control (PCT) [1], [2], [3]. LF is a disease caused by the lymphatic filarial roundworms *Wuchereria bancrofti*, *Brugia malayi* and *Brugia timori*, and transmitted by mosquitos. It is highly endemic in the tropics and subtropics (Africa, Asia, South Pacific and some parts of South America). The elimination strategy is through annual mass drug administration (MDA) with albendazole and ivermectin/diethylcarbamazine [1], [2]. LF elimination is implemented through the Global Programme to Eliminate Lymphatic Filariasis (GPELF) which has expanded MDA coverage from three million people treated in 12 countries in 2000, to more than 450 million in 53 countries in 2010 [4], [5]. During that period, the disease was eliminated in China and Korea. Nine countries no longer require MDA because of a natural decline in transmission intensity in areas of low disease endemicity. Globally, a total of 73 countries (including the recently independent Republic of South Sudan) are presently endemic for LF. Onchocerciasis, caused by *Onchocerca volvulus*, is transmitted by blackflies belonging to the *Simulium damnosum* complex. It is mainly endemic in Africa, Yemen and the Americas [6]. Control of the disease in Africa is through the African Programme for Onchocerciasis Control (APOC) using the annual community-directed treatment with ivermectin (CDTI) strategy [7]. In 2008 alone, 56.7 million people received treatment in 19 endemic African countries [7].

In Sierra Leone, both diseases are widely distributed across the country and co-endemic in 12 of the 14 health districts. The early distribution and clinical manifestations of both diseases in Sierra Leone were described in previous publications [8], [9], [10], [11], [12]. When Sierra Leone was included as part of the Onchocerciasis Control Programme (OCP) of WHO in 1989, treatment strategy for onchocerciasis control included aerial larviciding using helicopters and aircrafts targeting the breeding sites of the blackflies and ivermectin treatment as Merck & Co. Inc. had started donation of Mectizan (ivermectin) in 1987. National Onchocerciasis Control Programme (NOCP) records show that by 1994 annual biting rates of the savannah blackfly population dropped from the 1988 pre-treatment level of 60 bites/person/day to 1 bite/person/day and the community microfilaria load decreased by over 90%. However, by 1996 onchocerciasis control activities were stopped in all areas of the country when the civil conflict that started 1991 engulfed the entire country. The civil conflict ended in 2002, the same year that OCP was closed. NOCP activities recommenced in 2003 under the Special Intervention Zones (SIZ) established by APOC for some ex-OCP countries, including Sierra Leone. Surveys on onchocerciasis conducted in Sierra Leone after 2002 (unpublished NOCP data) showed that vector biting rates and community microfilaria load had reverted to pre-treatment levels in many communities. Since 2003 annual MDAs have been conducted for onchocerciasis control using the CDTI strategy with technical and financial support from APOC. The CDTI strategy, which promotes community participation as the key aspect of ivermectin distribution to improve access to ivermectin and ensure community ownership of the process, was adopted by APOC in the mid-1990s after a multi-country study. At first the local health workers and NGDO representatives introduce CDTI to the community in a participatory manner. Through a series of community meetings they explain the roles and responsibilities of communities in the CDTI process. The communities themselves then direct the planning and implementation of the interventions. The community collectively selects the community drug distributors (CDDs) and then plan the distribution process by deciding the method used (house to house or central location), the place where the distribution is conducted if fixed location is accepted, when the distribution is conducted, by whom activities will be implemented, how all activities will be monitored, and the support, if any, that CDDs will receive (financial or otherwise) from the community. With CDTI communities manage ivermectin by collecting their supply from a central point agreed upon with the health services and storing it within the community until the distribution period. The health workers and NGDO representatives train, supervise and monitor the CDDs while the community directs the process. It has been observed that when the community takes charge of onchocerciasis control MDAs can be sustained for up to 20 years. Furthermore, programme costs are reduced significantly because the community plays the leading role in all aspects of programme implementation [13], [14]. Apart from training of communities to assume leadership of the CDTI process, NGDOs have also made significant contribution to the CDTI process through operational research, provision of resources to complement national programmes by supporting health staff in remote communities, and provision of technical and financial support. An NDGO Coalition was created in 1991 for onchocerciasis control that meets regularly to coordinate collaboration at international and national levels [15]. Annual MDAs using the CDTI strategy has significantly reduced parasite prevalence and intensity in many communities of Sierra Leone since control operations resumed in 2003 [16]. Reports from health facilities had always indicated high endemicity of LF in all districts. Pre-baseline prevalence of LF was very high in south-eastern Sierra Leone. Blacklock (1922) examined 240 men in Mabang village and found 20% to be microfilaraemic, with prevalence of elephantiasis and hydrocoele of 4.6% and 3.8%, respectively [17]. Surveys in the early 1990s showed an average mf prevalence of 34.8% in three villages in the Moyamba district [18]. Similarly high prevalence rates were recorded in neighboring Liberia prior to the 1980s [19], [20]. In 2007–2008, the pre-treatment mf prevalence for the 12 districts outside the Western Area ranged from 0–6.9%, although prevalence was below 3% in the south-eastern districts [21] with Moyamba district showing pre-treatment mf prevalence of 1% (95%CI 0.4%–2.3%) [21]. This significant reduction of mf prevalence from earlier high levels prior to the start of the LF MDAs coincides with the commencement of mass administration of ivermectin for onchocerciasis control in the 1980s [16]. After national mapping of LF in 2005 and baseline data collection on microfilaria (mf) prevalence and density in 2007–2008 [21], CDTI was expanded to include albendazole distribution to control LF in six co-endemic districts in 2007 [16]. With support from the United States Agency for International Development (USAID) NTD Control Program, managed at the time by RTI International, the NOCP was transformed into the National Neglected Tropical Diseases Control Programme (NTDCP) in 2008 to upscale treatment for LF from 6 districts to all 14 endemic districts and integrate other NTDs such as schistosomiasis and soil transmitted helminthiasis into the control effort [16]. After the civil war in Sierra Leone, during which almost all health programmes had stopped, the Ministry of Health and Sanitation (MOHS) had decided to put the control of all NTDs under the existing onchocerciasis control programme with 1(one) programme manager responsible for all NTDs and working in close collaboration with strong district health management teams (DHMTs). It was decided that running vertical programmes for NTDs will be inefficient given the post war situation and the limited number of health workers and so the national coordination for NTDs had to work in close collaboration with the DHMTs and the existing district health structure.

Annual MDA with ivermectin and albendazole has been implemented since then. By early 2011, all 12 rural health districts (except Urban Western Area and Rural Western Area) had received at least three rounds of MDA. LF antigenemia prevalence (ICT) in 2005 was 11.7% (95% CI: 5.8%–22.2%) and 7.3% (95% CI: 3.1%–15.9%) for Urban Western Area and Rural Western Area respectively and baseline microfilaremia prevalence in 2008 was 0% (95% CI: 0%–0.7%) and 1.2% (95% CI: 0.6%–2.6%) for Urban Western Area and Rural Western Area respectively. The study presented in this manuscript is the midterm evaluation of the LF programme in Sierra Leone as part of the national NTD Control Programme and was conducted following guidelines provided by WHO, which recommends midterm programme review before the 4^th^ round of MDA. The 2 LF-only districts were not included in this study because effective MDA in these 2 districts started in 2010, while effective MDA in the other districts started in 2007/2008. These 2 districts have been treated through MDAs since 2010 but post-MDA microfilaremia studies have not yet been done. According to WHO guidelines [22], a mid-term survey was conducted in July/August 2011 in sentinel and spot check sites in the 12 rural health districts. The hypothesis of the study is that areas previously exposed to ivermectin treatment for onchocerciasis control may require fewer rounds of MDA to interrupt transmission of LF. Study objectives are to assess midterm progress towards LF elimination by measuring the microfilaremia prevalence for LF in districts that had conducted 3 good round of MDA and identify any implementation units (districts) that may require additional effort to reach the target of LF elimination. In this paper we describe the impact of three rounds of MDA on LF prevalence and mf density in areas of low LF endemicity which may be related to previous treatment with ivermectin for onchocerciasis control.

## Methods

### Ethics Statement

This study was conducted by the National NTDCP of the MOHS, Sierra Leone as part of the routine monitoring and evaluation activities of the national control programme. Ethical approval for the study was obtained from the MOHS Research and Ethics Committee. Informed oral consent was obtained from each participant before samples were collected. Parents and guardians provided informed consent for child participants to participate in the study before samples were collected. The acceptance of all participants/parents and guardians (for children) was recorded on a form by the team leader, as literacy rates are low in the country. All participants aged 5 years and above in each site were eligible for inclusion without discrimination on gender, social status, religion or ethnicity. Participants' identities were protected by collecting, recording and analyzing data such that participants remained anonymous.

### Mass Drug Administration

Annual MDA with ivermectin and albendazole was piloted in 2007 in six rural districts located in border areas with neighboring Guinea and Liberia: Bombali, Kailahun, Kambia, Koinadugu, Kono, and Pujehun. This was scaled up to cover 12 rural districts in 2008 with six additional districts added to the previous six: Bo, Bonthe, Kenema, Moyamba, Port Loko and Tonkolili. Geographic coverage for the endemic districts targeted reached 100% in 2010 when MDA was scaled up to cover the remaining two health districts: Urban Western area and Rural Western area [23]. Within rural communities ivermectin and albendazole were distributed by CDDs who are literate members of the respective communities selected by their communities and trained by health workers. CDDs are trained by district health workers to conduct pre-MDA census, house-to-house visits in the village, treat all eligible members of the community by observing them while they take the doses, conduct follow up visits to treat absentees and complete the relevant reporting tools used at community level. 1 CDD is trained to cover approximately 100 people and for Sierra Leone where the average population per community is about 200, each community has on average 2 CDDs. In urban areas the programme tried but could not succeed in getting community volunteers (CDDs) to distribute the ivermectin and albendazole without getting any financial payment as in rural areas and so students in medical and nursing institutions were trained and paid to conduct MDAs. District health workers conduct trainings for MDA and provide supervision during MDAs. NTDCP staff and members of the DHMTs also supported training and supervision for MDAs. MDA is conducted once a year between September and December, which is the post-harvest period that communities have accepted for MDAs.

Before each MDA, CDDs conduct a pre-MDA census. Details on all community members are recorded in the community registers and updated each year prior to subsequent MDA. MDA details are also captured in the registers. After each MDA, details are summarized in the reporting forms by drug distributors and submitted to the supervising health workers. The supervising health workers prepare summary reports for all villages/urban areas targeted and submit the reporting forms to the DHMTs. Each DHMT then submits the district MDA report to the NTDCP, which collates MDA results from all districts. It should be noted that all activities were co-implemented for both onchocerciasis and LF control starting from trainings of district health workers and CDDs, community sensitization and mobilization, advocacy and mass distribution of ivermectin and albendazole. The NTD control programme is also strongly integrated in the national and district health system and has benefitted from a well-structured health system at district level that has a focal person responsible for NTD control within each district, which ensures high treatment and geographic coverage.

MDA in the 6 districts that piloted MDA for LF in 2007 took place in rural areas (villages) only as the main aim of this pilot MDA was to see how the CDDs and the district health workers can manage integrated MDA for onchocerciasis and LF (i.e. distribution of both ivermectin and albendazole). The onchocerciasis control programme is not implemented in urban areas with large populations or populations greater than 2000 people. Therefore, the integrated MDA in 2007 was done only in areas previously treated for onchocerciasis. As the 6 districts that piloted MDA for LF in 2007 did not cover the urban areas (i.e. district headquarter towns and other large towns with population >2000 people) with relatively poor treatment coverage (well below 65%), the 2007 MDA results were considered inadequate. It was only in 2008 that urban areas of the 12 districts were treated using health workers as distributors. 2008 is therefore considered year 1 when MDA results were “adequate” as treatment coverage was above 65% and geographic coverage was 100%. Please see tables 1 and 2 for districts that conducted pilot MDA in 2007.

**Table 1:** LF microfilaraemia prevalence/density at baseline and midterm, their percentage reductions and p-values.*

	Baseline survey				Mid-term assessment				Percentage reduction			Significance test for reduction (p values)		
	No of persons examined for Mf	Mf prevalence (%) (95% CI)	Population mf density (mf/ml) (95% CI)	Positive-only mf density (mf/ml) (95% CI)	No of persons examined for Mf	Percentage mf prevalence (95% CI)	Population mf density (mf/ml) (95% CI)	Positive-only mf density (mf/ml) (95% CI)	Mf prevalence	Population mf density	Positive-only mf density	Mf prevalence	Population mf density	Positive-only mf density
**Overall**	8233	2.6 (2.3–3.0)	1.32 (1.00–1.65)	50.9 (40.25–61.62)	6023	0.30 (0.19–0.47)	0.05 (0.03–0.08)	17.59 (15.64–19.55)	88.5	96.2	65.4	0.000	0.000	0.001
***By district***														
Bo	1005	2.0 (1.3–3.1)	1.97 (0.84–3.11)	99.17 (58.32–140.01)	500	0	0	0	100	100	100	0.001	0.002	-
** Bombali	830	6.9 (5.3–8.8)	1.93 (1.28–2.57)	28.07 (21.70–34.44)	506	1.58 (0.80–3.09)	0.26 (0.08–0.45)	16.67 (-)	77.1	86.3	40.6	0.000	0.000	0.068
Bonthe	504	1.2 (0.6–2.6)	0.83 (0.02–1.63)	69.44 (13.68–125.21)	499	0.20 (0.04–1.13)	0.03 (0–0.10)	16.67 (-)	83.3	96.0	76.0	0.060	0.059	0.295
**Kailahun	624	2.6 (1.6–4.1)	2.08 (0.00–4.89)	81.25 (0.00–195.58)	499	0.20 (0.04–1.13)	0.03 (0–0.10)	16.67 (-)	92.3	98.4	79.5	0.001	0.001	0.472
**Kambia	619	2.1 (1.2–3.6)	0.97 (0.23–1.71)	46.15 (17.04–75.27)	500	0.40 (0.11–1.45)	0.07 (0–0.16)	16.67 (-)	81.0	93.1	63.9	0.014	0.014	0.311
Kenema	1016	0.6 (0.3–1.3)	0.34 (0.00–0.70)	58.33 (4.42–112.24)	500	0	0	0	100	100	100	0.085	0.085	-
**Koinadugu	636	5.7 (4.1–7.7)	1.99 (0.95–3.04)	35.19 (19.83–50.54)	498	0.80 (0.31–2.05)	0.17 (0–0.34)	20.83 (7.57–34.09)	86.0	91.6	40.8	0.000	0.000	0.454
**Kono	875	2.4 (1.6–3.6)	1.11 (0.37–1.84)	46.03 (20.09–71.97)	499	0	0	0	100	100	100	0.000	0.000	-
Moyamba	500	1 (0.4–2.3)	0.67 (0.00–1.36)	66.67 (6.33–127.00)	500	0	0	0	100	100	100	0.025	0.025	-
Port Loko	500	4.4 (2.9–6.6)	3.53 (1.48–5.59)	80.30 (44.49–116.12)	499	0.20 (0.04–1.13)	0.03 (0–0.10)	16.67 (-)	95.5	99.1	79.2	0.000	0.000	0.219
**Pujehun	624	0 (0–0.6)	1.19 (0.90–1.48)	-	500	0	-	-	-	-	-	-	-	-
Tonkolili	500	2.4 (1.4–4.2)	0.63 (0.24–1.03)	26.39 (17.99–34.79)	523	0.19 (0.03–1.08)	0.03 (0–0.10)	16.67 (-)	92.1	94.9	36.8	0.002	0.002	0.442
***By sex***														
Male	3863	3.3 (2.8–3.9)	1.83 (1.21–2.44)	55.08 (39.00–71.15)	3170	0.35 (0.19–0.62)	0.06 (0.03–0.10)	18.18 (14.80–21.56)	89.4	96.7	67.0	0.000	0.000	0.013
Female	4370	2.0 (1.6–2.4)	0.88 (0.59–1.18))	44.76 (32.89–56.64)	2853	0.25 (0.12–0.51)	0.04 (0.01–0.07)	16.67 (-)	87.5	95.4	62.8	0.000	0.000	0.023
***By age groups***														
5–14	-	-	-	-	1947	0.21 (0.08–0.53)	0.04 (0–0.09)	20.83 (7.57–34.09)	-	-	-	-	-	-
15–20	1614	2.0 (1.4–2.8)	0.90 (0.43–1.37)	45.31 (26.73–63.89)	858	0.12 (0.02–0.66)	0.02 (0–0.06)	16.67 (-)	94.0	97.8	63.2	0.000	0.000	0.341
21–30	1750	2.8 (2.1–3.7)	2.00 (0.81–3.19)	71.43 (32.45–110.41)	858	0.58 (0.25–1.36)	0.10 (0.01–0.18)	16.67 (-)	79.3	95.0	76.7	0.000	0.000	0.042
31–40	1623	2.6 (2.0–3.6)	1.14 (0.62–1.66)	43.02 (27.54–58.50)	849	0.59 (0.25–1.37)	0.10 (0.01–0.18)	16.67 (-)	77.3	91.2	61.3	0.000	0.000	0.059
41–50	1271	3.6 (2.7–4.8)	1.72 (0.99–2.44)	47.46 (32.02–62.90)	640	0.47 (0.16–1.37)	0.08 (0–0.17)	16.67 (-)	86.9	95.3	64.9	0.000	0.000	0.159
>50	1975	2.2 (1.7–3.0)	0.97 (0.53–1.41)	43.56 (27.68–59.44)	871	0	0	0	100	100	100	0.000	0.000	-

* The table shows crude mf prevalence and mf density by district, sex and age group, their percentage reductions and significance test for reductions of mf prevalence and density after 3 rounds of MDA in Sierra Leone.

** Districts that piloted MDA in 2007.

**Table 2:** Summary results of annual MDA*** for LF**** elimination in Sierra Leone 2008–2010.

			2008					2009					2010				
Districts	Villages/Urban areas targeted*	Villages/Urban areas treated	Total pop. of IUs	Eligible pop. of IUs	Total treated in IUs	Prog. Cov. by IUs	Drug cov. by IUs	Total pop. of IUs	Eligible pop. of IUs	Total treated in IUs	Prog. Cov. by IUs	Drug cov. by IUs	Total pop. of IUs	Eligible pop. of IUs	Total treated in IUs	Prog. cov. by IUs	Drug cov. by IUs
Bo	1367	1367	574053	487945	380676		78.0	595318	506020	420968	70.7	83.2	613178	521201	445996	72.7	85.6
** Bombali	1596	1596	440932	374792	316672	71.8	84.5	454604	386413	350278	77.1	90.6	498115	423398	363078	72.9	85.8
Bonthe	550	550	166140	141219	98856	59.5	70.0	150718	128110	110834	73.5	86.5	154860	131631	117201	75.7	89.0
**Kailahun	977	977	392819	333896	287536	73.2	86.1	401215	341033	313367	78.1	91.9	410509	348933	322206	78.5	92.3
**Kambia	837	837	269673	229222	202999	75.3	88.6	289136	245766	211926	73.3	86.2	310705	264099	234910	75.6	88.9
Kenema	1380	1380	551797	469027	391778	71.0	83.5	601661	511412	439136	73.0	85.9	583278	495786	449763	77.1	90.7
**Koinadugu	1041	1041	207995	176796	151395	72.8	85.6	216472	184001	157339	72.7	85.5	222966	189521	162059	72.7	85.5
**Kono	1360	1360	466223	396290	321833	69.0	81.2	442235	375900	323907	73.2	86.2	461562	392328	346719	75.1	88.4
Moyamba	1539	1539	309436	263021	232327	75.1	88.3	304416	258754	232859	76.5	90.0	350779	298162	268876	76.7	90.2
Port Loko	1769	1769	376212	319780	250457	66.6	78.3	547672	465521	386929	70.6	83.1	480920	408782	363026	75.5	88.8
**Pujehun	813	813	261509	222283	188872	72.2	85.0	272436	231571	210954	77.4	91.1	250280	212738	193485	77.3	90.9
Tonkolili	1024	1024	368678	313376	252785	68.6	80.7	418828	356004	318229	76.0	89.4	412404	350543	304195	73.8	86.8
	**14253**	**14253**	**4385467**	**3727647**	**3076186**	**70.1**	**82.5**	**4694711**	**3990504**	**3476726**	**74.1**	**87.1**	**4749556**	**4037123**	**3571514**	**75.2**	**88.5**

* Geographic coverage of villages and urban areas was 100% in all 12 districts over the 3 years (2008–2010).

** Districts that piloted MDA in 2007.

*** MDA = mass drug administration.

**** LF = lymphatic filariasis.

### Survey Site Selection

34 Villages were randomly selected by AFRO in Brazzaville using the available database for villages in Sierra Leone in 2005 with at least 2 villages selected per district depending on the population and sent to the programme. After the mapping in 2005, villages with relatively very high antigenemia prevalence were selected for all 14 health districts as sentinel sites for the baseline mf survey in 2007/2008. The number of sentinel sites selected per district depended on the population of the district. The then WHO guidelines recommended 1 sentinel site per 500,000 population and 1 sentinel site was selected for districts with population less than 500,000 and 2 for districts with population more than 500,000 [21], [24].

Sampling for the midterm survey July/August 2011 was conducted in accordance with new WHO guidelines in one sentinel site and one spot check site per population of one million people [22]. The 12 rural districts that had conducted at least three rounds of MDA were involved in this study. As the populations of the districts were small, the 12 districts were put in six groups of two districts depending on geographical proximity and epidemiological characteristics so that the total population for each group was about a million [22]. In each of the six groups (table 3), a sentinel site was selected in one district for this study, and a spot check site was selected in the other district, in consultation with the DHMTs. The groups included the following pairs of districts: Bonthe (sentinel site (SS)- Moboya) and Moyamba (spot check site (SCS)- Taninahun Kapuima); Koinadugu (SS-Kumala) and Bombali (SCS-Makoba Yelima); Bo (SS-Gelehun) and Pujehun (SCS- Kundorwahun); Port Loko (SS-Gbabai) and Kambia (SCS- Kamasasa); Kailahun (SS-Manowa) and Kenema (SCS- Joru); Kono (SS- Tombodu) and Tonkolili (SCS-Rosint). In the “sentinel site” districts data obtained in this study were compared with baseline data, while among the “spot check site” districts, the results of this survey were compared with baseline results obtained in the original sentinel sites in these districts.

**Table 3:** Survey site selection.

Groups of districts	Districts	Sentinel sites	Spot check sites
1	Bonthe	Moboya	-
	Moyamba	-	Taninahun Kapuima
2	Koinadugu	Kumala	-
	Bombali	-	Makoba Yelima
3	Bo	Gelehun	-
	Pujehun	-	Kundorwahun
4	Port Loko	Gbabai	-
	Kambia	-	Kamasasa
5	Kailahun	Manowa	-
	Kenema	-	Joru
6	Kono	Tombodu	-
	Tonkolili	-	Rosint

The spot check sites were selected in consultation with DHMTs because according to WHO guidelines of 2011 spot check sites are to be selected according to the local knowledge where LF is most likely to be found as the objective of LF control is elimination [22]. By consulting with DHMTs and selecting areas where LF prevalence could be high the possibility of selecting spot check sites that will have zero prevalence while there were areas with high prevalence within the same districts might have been avoided [22].

Recent WHO guidelines [22] recommend study of a minimum of 300 participants per sentinel/spot check site but villages in Sierra Leone generally have small populations (average of 250) and so in most cases all those 5 years and above that volunteered in the sentinel/spot check villages were simply selected while others in neighboring villages were randomly selected to have a number greater than 300 participants. WHO recommends convenience sampling for any group selected for LF survey because they are seen to be at high risk [22].

### Sampling and Diagnosis

The survey teams met with community leaders upon arrival in communities and explained the nature of their work, after which, meetings were held with the general community to explain the study and its significance and respond to questions from community members before the study was conducted. Some 300–500 participants of 5 years of age or above were recruited per site according to WHO guidelines [22]. In sites with less than 300 participants, more participants were recruited in neighboring villages. To ensure standardization of activities and data, two-day practical training was conducted for all technicians before the study started. Fingertip blood was collected between 10 pm and 2 am. A 60 µl blood sample was collected from each participant, smeared gently and uniformly in a circular shape and allowed to air dry at room temperature for 12–24 hours. The next day, the dried smear was dehaemoglobinized through flooding with distilled water for 3–5 minutes, air dried again, fixed with methanol for 30–60 seconds, stained with GIEMSA for 10 minutes, and examined for mf under a light microscope by experienced examiners. The ×40 objective was used to first locate the mf by moving patiently from left to right or right to left starting at the extreme top end of the thick blood film and moving through all available fields; then moving slightly downwards and repeating the same process of moving from left to right or right to left until all areas of the thick blood film are covered. When mf is located the filarial species was identified using ×100 objective [22]. A minimum of 50 microscopic fields were examined before a specimen was considered negative. The research team included laboratory technicians from the national reference laboratory and the University of Sierra Leone who have adequate experience in diagnostic detection of filarial parasites. The team leader was also supported in 2007 by WHO and the NTD Support Center in Ghana to receive further training on detection of filarial parasites at the Noguchi Memorial Institute for Medical Research in Accra, Ghana. Positive findings of mf were recorded and individual mf density of infection was calculated and expressed as the number of mf per ml of blood (mf/ml). A total of 6,023 “midnight” blood samples were collected and examined for mf as shown in table 1, male 3,170 (52.6%) and female 2,853 (47.4%). The mean age (± standard deviation) of the subjects examined was 28.91±18.92 years (males: 27.65±18.77, females: 30.32±18.92). For quality control, all positive slides and 10% of the negative slides were preserved and examined by a researcher, who was invited during the design of the study to help in designing the study and to conduct the quality control because he has been involved in the study and detection of filarial parasites since 1995–1996 [18]. There were only 18 positive slides and these were submitted for quality control together with 600 randomly selected negative slides. Results of the quality control showed that all 18 positive slides were true positives while the negative slides were all true negatives. The coordinates of each sample site were recorded using hand-held units of global positioning system (site coordinates available upon request).

### Statistical Analysis

Results were entered into MS Excel and analyzed in SPSS (IBM, Version 19). Prevalence and density of mf were calculated for all 12 districts and compared with the baseline data. The 95% confidence intervals (CIs) for prevalence were calculated using the Wilson score method without continuity correction [25]. The arithmetic mean mf density of infection with 95% CI was calculated using the total population examined and the positive samples only [21], [24]. The Chi-squared test was used to compare the differences in prevalence and the Kruskal-Wallis test was used to compare the differences in mf density. Treatment coverage was calculated according to the WHO guidelines [22]. Epidemiological drug coverage (EDC), otherwise known as Programme coverage, is the treatment coverage reported using total population of IU as denominator and is calculated as the number of people who were reported to have ingested the medicines for LF divided by total population in IU multiplied by 100. The epidemiological drug coverage calculated using the total population of the IU is a reflection of what proportion of the at-risk population is being covered by MDA. Drug coverage (DC) is the treatment coverage reported using individuals targeted or eligible for treatment in the IU as denominator and is calculated as the number of people who were reported to have ingested the medicines for LF divided by all individuals targeted or eligible for treatment in the IU multiplied by 100. The drug coverage in the targeted or eligible population is considered the best measure of how well MDAs are implemented. An adequate level of EDC is estimated to be 80% and the DC should be close to 100%. These indicators enable IU authorities to assess the status of the elimination programme. WHO recommends that programme managers use the reported coverage to identify areas with low coverage, investigate the causes and find solutions that will improve programme implementation as the programme continues [22]. The total population for rural areas used as denominator for analyzing MDA results was the total number of people registered during the pre-MDA census, while the total population used in urban/non-rural areas was the projected figure according to the 2004 national census [26], with an annual growth rate of 2.5%. Spatial analysis of the LF mf prevalence was conducted using the kriging method in the Geostatistical Analyst Extension of ArcGIS version 10 (ESRI, Redlands, USA). Spatially smoothed contour maps of the interpolated prevalence of mf at baseline and after three MDAs were produced as described previously [21], [27].

## Results

### Mass Drug Administration Results 2008–2010

A total of 14,253 villages and urban areas were treated for LF each year during the 3 years in the 12 districts. As all the villages and urban areas were treated in each of the 12 districts, this represents 100% geographic coverage for endemic villages and urban areas in all 12 districts during each of these 3 rounds of MDA, as shown in table 2. Over 4 million people were targeted for treatment each year during the 3 years. Overall EDC was 70.1%, 74.1% and 75.2% in 2008, 2009 and 2010 respectively at the national level, and was ≥65.0% in each district in each round, except in Bonthe, where it was 59.5% in 2008. EDC also improved between 2008 and 2010. Five districts had <70.0% in 2008 (Bo: 66.3%, Bonthe: 59.5%, Kono: 69.0%, Port Loko: 66.6% and Tonkolili: 68.6%); while in 2009 and 2010, all districts had >70.0% EDC, as shown in table 2. The overall DC was 82.5%, 87.1% and 88.5% in 2008, 2009 and 2010, respectively. The DC is a measure of how well MDA was conducted and is considered adequate when ≥80.0% [22]. DC by district in each round was ≥80.0%, except in Bo, Bonthe and Port Loko, which had 78.0%, 70.0% and 78.3% respectively in 2008, as shown in table 2.

### Microfilaraemia Prevalence

Five districts (Bo, Kenema, Kono, Moyamba and Pujehun) had 0.0% mf prevalence. One district (Pujehun) had baseline mf prevalence of 0.0%, which was maintained. Another six districts had mf prevalence between 0.0 and 1.0%: Bonthe (0.20%; 95% CI: 0.04%–1.13%), Kailahun (0.20%; 95% CI: 04%–1.13%), Kambia (0.40%; 95% CI: 0.11%–1.45%), Koinadugu (0.80%; 95% CI: 0.31%–2.05%), Port Loko (0.20%; 95% CI: 0.04%–1.13%) and Tonkolili (0.19%; 95% CI: 0.03%–1.08%). Only one district had mf prevalence of over 1%: Bombali (1.58%; 95% CI: 0.80%–3.09%). Overall mf prevalence among males was 0.35% (95% CI: 0.19%–0.62%), and among females 0.25% (95% CI: 0.12%–0.51%). Prevalence by age group, 5–14 years (N = 1947), 15–20 years (N = 858), 21–30 years (N = 858), 31–40 years (N = 849) and 41–50 years (N = 640), was 0.21% (95% CI: 0.08%–0.53%), 0.12% (95% CI: 0.02%–0.66%), 0.58% (95% CI: 0.25%–1.36%), 0.59% (95% CI: 0.25%–1.37%) and 0.47% (95% CI: 0.16%–1.37%) respectively, while prevalence in the age group >50 years (N = 871) was 0.0%. In total, 18 persons (0.30%, 95% CI: 0.19–0.47%) had a positive blood smear, and there was no significant difference in mf prevalence in males as compared to females (p = 0.47). There were also no significant differences in prevalence among age groups.

**Figure 1:**
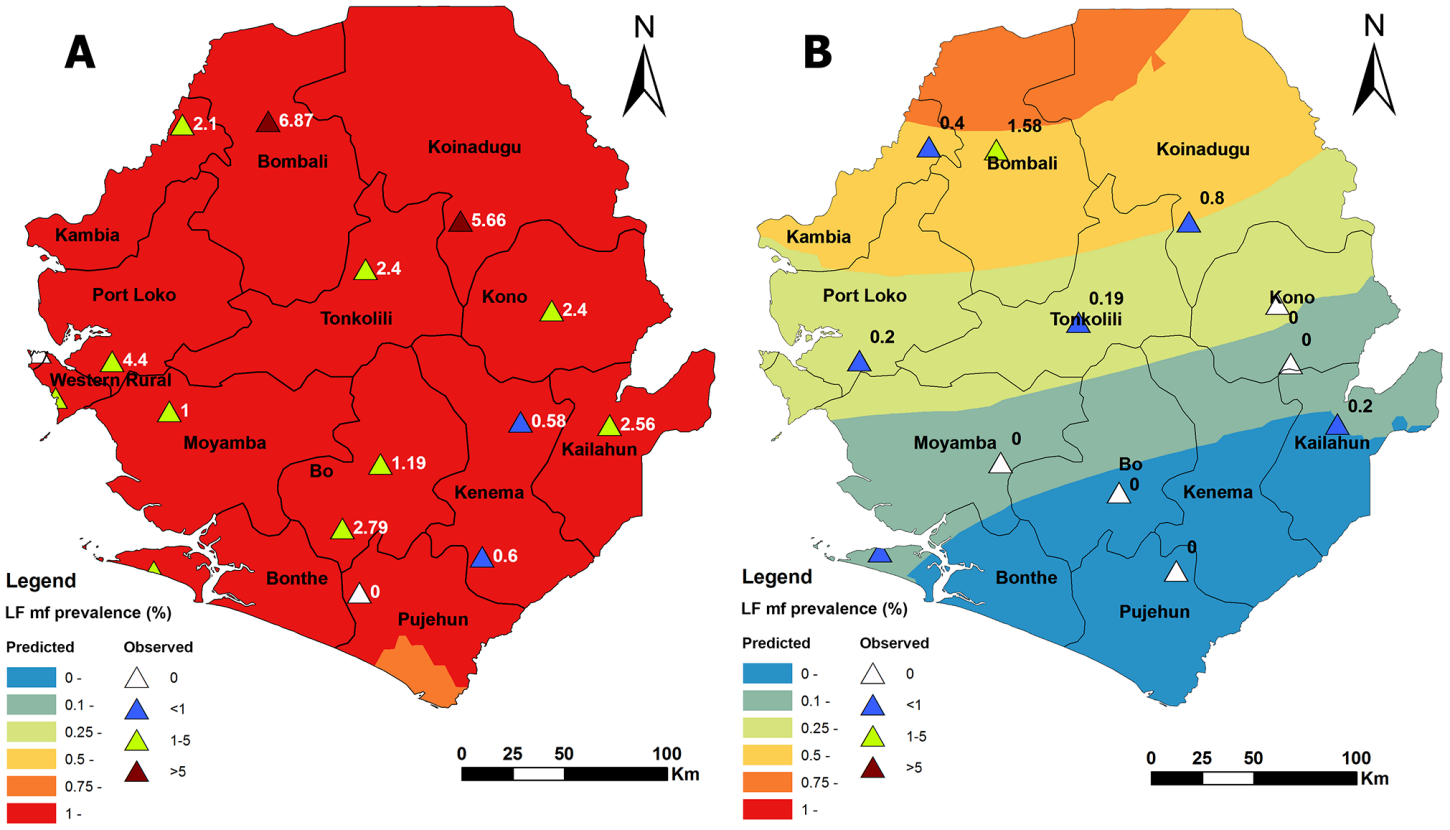
Survey sites and spatially smoothed contour maps of predicted LF mf prevalence in Sierra Leone. **A**. Predicted mf prevalence at baseline; **B**. Predicted mf prevalence after three rounds of MDA. The same legend scale was used for the contour map of both A and B for easy comparison. Triangles and labels show the survey locations and the observed mf prevalence in each location.

**Figure 2:**
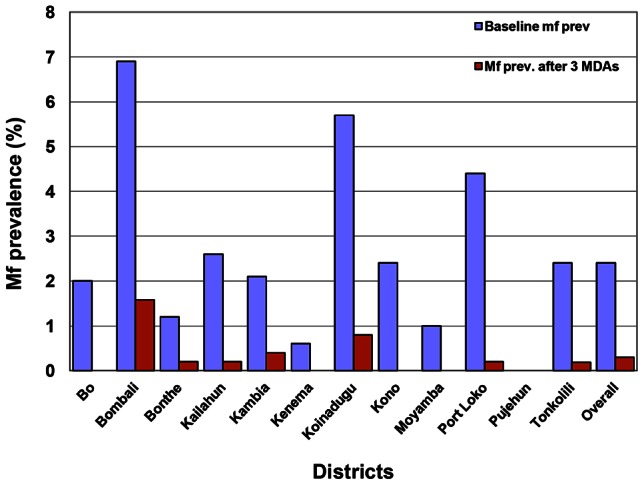
Reduction of MF prevalence after 3 annual MDAs for LF in Sierra Leone 2008–2010.

Compared with the baseline, overall mf prevalence decreased by 88.5% (p = 0.000), from 2.6% (95% CI: 2.3%–3.0%) to 0.30% (95% CI: 0.19%–0.47%), after 3 rounds of MDA. As shown in table 1, among the 11 districts with baseline mf prevalence ≥1%, seven districts showed mf prevalence reduction of over 90% after three rounds of MDA, three districts by over 80%, and only one district by below 80%. Spatial prediction suggested a sweeping reduction in mf prevalence from the baseline level after three MDAs across the country. There was an 89.4% decrease (p = 0.000) in mf prevalence among males: 3.3% (95% CI: 2.8%–3.9%) to 0.35% (95% CI: 0.19%–0.62%); and an 87.5% decrease (p = 0.000) in mf prevalence among females: 2.0% (95% CI: 1.6%–2.4%) to 0.25% (95% CI: 0.12%–0.51%). There was 0.21% (95% CI: 0.08%–0.53%) prevalence among the age group 5–14 years, but this could not be compared, as the baseline study did not include participants <15 years. Decreases in mf prevalence among the age groups 15–20, 21–30, 31–40, 41–50 and >50 years ranged between 77.3% and 100.0% (p = 0.000, 0.000, 0.000, 0.000 and 0.000 respectively). Figure 1 shows predicted mf prevalence at baseline (A) and predicted mf prevalence after three rounds of MDA (B). Figure 2 shows the overall decrease in mf prevalence and the decrease for each district. A statistical comparison between the 6 districts that piloted MDA for LF in 2007 and the other 6 showed no statistical difference between the decreases in microfilaremia prevalence of the 2 groups of districts. 3 out of the 4 districts that had 100% decreases in mf prevalence had conducted only 3 MDAs.

### Microfilaraemia Density

**Figure 3:**
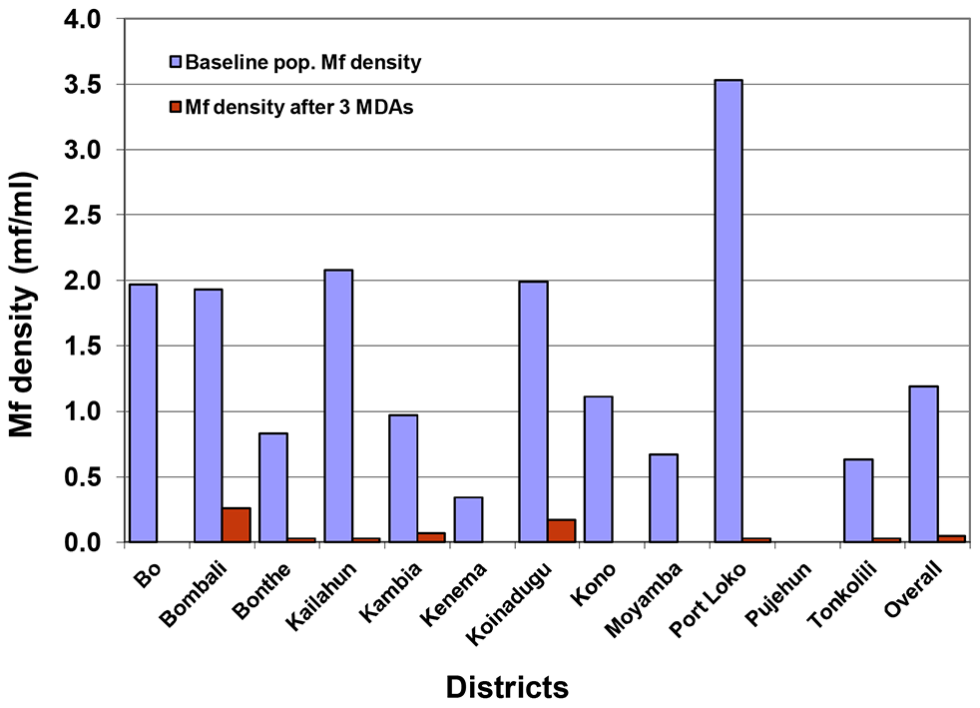
Reduction of entire-population mf density after 3 annual LF MDAs in Sierra Leone 2008–2010.

**Figure 4:**
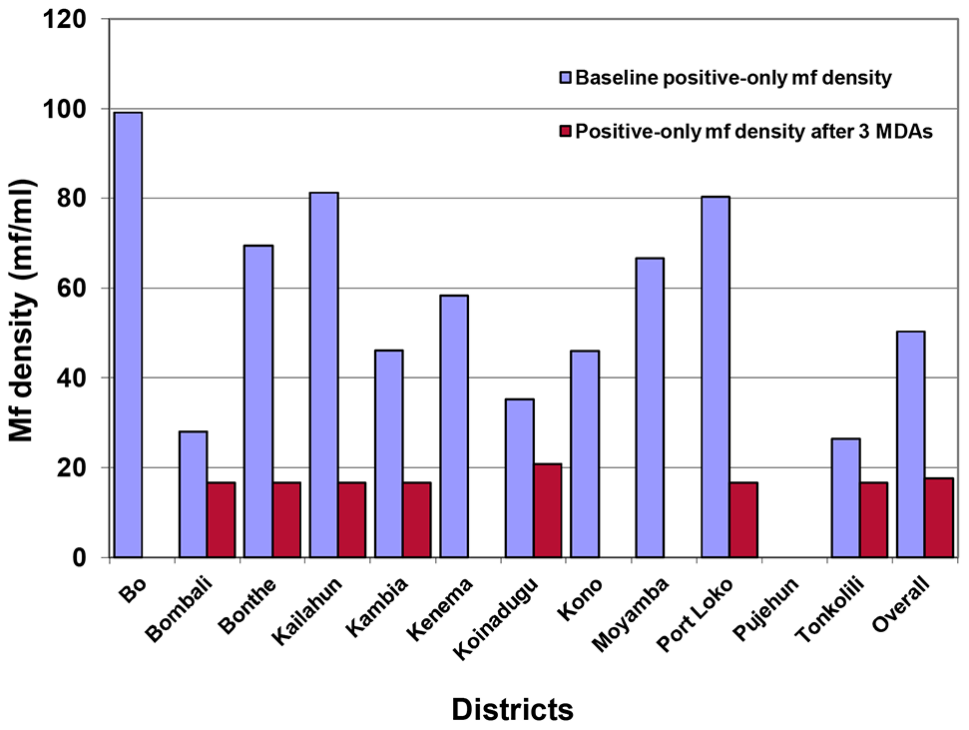
Reduction of positive-only mf density after 3 annual LF MDAs in Sierra Leone 2008–2010.

The overall arithmetic mean mf density was 0.05 mf/ml (95% CI: 0.03 mf/ml–0.08 mf/ml) in the total participants examined and 17.59 mf/ml (95% CI: 15.64 mf/ml–19.55 mf/ml) among mf-positive individuals. The mean mf density by district was well below 1 mf/ml for the population examined and below 21 mf/ml among those who were mf positive. There was no significant difference in mf density in males versus females (p>0.05). There was also no significant difference in mf density among age groups in the total population examined (p>0.05). Overall mean mf density among mf positive individuals decreased by 65.4% (p = 0.001), from 50.9 mf/ml (95% CI: 40.25 mf/ml–61.62 mf/ml) at baseline to 17.59 mf/ml (95% CI: 15.64 mf/ml–19.55 mf/ml); and in the total population examined, there was a 96.2% decrease (p = 0.000), from 1.32 mf/ml (95% CI: 1.00 mf/ml–1.65 mf/ml) at baseline to 0.05 mf/ml (95% CI: 0.03 mf/ml–0.08 mf/ml). In Bo, Kenema, Kono and Moyamba, there was 100.0% decrease in mf density among both mf positive participants and the entire population. Six districts, Bonthe, Kailahun, Kambia, Koinadugu, Port Loko and Tonkolili, had a >90.0% decrease in mf density for the entire population (p = 0.059, 0.001, 0.014, 0.000, 0.000 and 0.002 respectively), and a >36.0% decrease in mf density among positive participants (p = 0.295, 0.472, 0.311, 0.454, 0.219 and 0.442 respectively). Bombali had the lowest decreases in mf density, 86.3% for the entire population (p = 0.000) and 40.6% among positive individuals (p = 0.068). Table 1 shows the reduction of mf density in the 12 districts after 3 MDAs. There was a 96.7% decrease in mf density among all males (p = 0.000) and a 67.0% decrease in mf density among males that are mf positive (p = 0.013); and there was a 95.4% decrease in mf density among all females (p = 0.000) and a 62.8% decrease in mf density among females that are mf positive (p = 0.023). The age groups 15–20, 21–30, 31–40 and 41–50 years had >90.0% decrease in mf density for the entire population (p = 0.000, 0.000, 0.000 and 0.000 respectively) and >60.0% decrease in mf density among mf positive individuals (p = 0.341, 0.042, 0.059 and 0.159 respectively). The age group >50 years had a 100.0% decrease in mf density for the entire population and among mf positive individuals. For details of mf prevalence and density at baseline and after 3 MDAs, reductions in mf prevalence and density after 3 MDAs and p values for the reductions in prevalence and density please see table 1. Figures 3 and 4 show overall and district decreases in mf density for the entire population and for those who were mf positive respectively.

## Discussion

LF is widely endemic across Sierra Leone, transmitted by *Anopheles* mosquitoes. All 14 health districts qualified for MDA intervention in accordance with WHO guidelines because they had baseline LF prevalence by ICT cards ≥1.0% [21], [24]. Although MDA was piloted in rural areas of 6 health districts in 2007, the 2007 MDA results were relatively poor and considered “inadequate” and so 2008 is considered year 1 for LF MDA when treatment and geographic coverage was ≥65% and 100% respectively. The results from the 12 rural districts showed that over the three years (2008–2010), geographic coverage was 100% in all 12 districts, EDC was ≥65.0% in all districts except for Bonthe in 2008 (59.5%), and DC was ≥80.0% in all districts except for Bo (78.0%), Bonthe (70.0%) and Port Loko (78.3%) in 2008. The treatment coverage was verified through independent monitoring activities, as described previously [23]. The current assessment showed that the average mf prevalence in the country was only 0.30% and the average population mf density was only 0.05 mf/ml after three rounds of MDA, with no microfilaria detected in six of the 12 districts, including all the districts in the Southern Province and only one district showing mf prevalence of >1% (Bombali, 1.58%). This represents an overall reduction of 87.5% in mf prevalence and 95.5% in population mf density. The zero mf prevalence recorded for Pujehun district at baseline may have been as a result of the randomness of the selection of the sentinel sites. Consequently, a spot check site was selected in Pujehun for the midterm study in consultation with the DHMT of Pujehun district based on results of reported hydroceles and lymphedema, which increased the chances of finding mf positive cases. Since the mf prevalence is again zero it is recommended that another spot check site be selected for the next survey in the district (the pre-transmission assessment survey). The use of pre-MDA census data as denominator in rural settings versus use of projected census population as denominator in urban or non-rural settings for the calculation of MDA results may have created bias in terms of interpretation and comparability of MDA results. However, it should be noted that the issue of what denominator to use for MDAs in urban settings still has to be resolved by the international NTD community as this poses a big challenge for national control programmes. Pre-MDA census in urban settings could be cumbersome, very expensive and results reported cannot be easily validated. The NTDCP therefore decided to use projected census figures as denominator in the analysis of MDA results for non-rural or urban areas of the districts.

The number of MDA rounds needed to eliminate LF depends on baseline infection rates, vectoral capacity, efficacy of the MDA regimen used, and community compliance with treatment. It is possible to eliminate LF in some implementation units (IUs) with low baseline infection rates using less than five rounds of MDA, while more than six MDA rounds may be needed for IUs with relatively high baseline LF prevalence [28], [29], [30]. The high level of reduction in mf prevalence and intensity after three rounds of MDA in Sierra Leone may have been partly due to the relatively low baseline mf level [21].

Several studies on LF conducted before the baseline studies in 2007/2008 in Sierra Leone and neighboring Liberia show mf prevalence ≥20% but the LF prevalence at baseline (2007/2008) ranged from 0%–6.9% for all districts with prevalence of the southeastern districts that were studied previously <3% at baseline [21]. Many studies have shown that there are 3 drugs that have microfilaricidal effect on the lymphatic filarial roundworms and are available for LF treatment: diethylcarbamazine (DEC), ivermectin and albendazole. Treatment with DEC or ivermectin alone significantly reduces blood mf levels (up to 90% mf clearance is reported) but combination of both drugs is more effective than using one drug. The marked filaricidal effect of these drugs makes them suitable for annual treatment designed to control transmission immediately and in the long term to control morbidity [31]. In Burkina Faso and India it was demonstrated that 5–14 years of ivermectin treatment (i.e. treating with ivermectin alone) reduced mf prevalence and intensities of W. bancrofti but transmission was not interrupted. The treatment rounds with ivermectin alone can significantly reduce prevalence and intensity of W. bancrofti microfilaremia, which provides an opportunity for synergy where onchocerciasis and LF are coendemic [32], [33], [34]. It is reported that there is a strong relationship between mf prevalence and intensity in humans and mf intake and development in the mosquito vector which means that lower intensity can lead to reduced transmission [34], [35]. The mass administration of ivermectin for onchocerciasis control using the CDTI strategy, which has been demonstrated to be very effective in reaching the target communities and populations, could have been responsible for the reduction in mf prevalence and density at baseline as indicated in previous studies mentioned above. The reduction in mf prevalence as a result of ivermectin treatment could have resulted in reduced transmission among the populations of the 12 districts because mf intake and development within the vector depends on the level of mf prevalence and density. Low mf prevalence and density could have resulted in reduced mf intake and development in the mosquito vector, reduced mf transmission and therefore even further reduction of mf prevalence and density in the populations with time.

By studying infection and infectivity prevalence in the vector mosquitos it was demonstrated in Nigeria that 5 years of semi-annual MDAs with ivermectin alone targeted at onchocerciasis control reduced but did not interrupt transmission of W. bancrofti [36], [37]. Adding albendazole provided better mf clearance (up to 99%) and clearance of soil transmitted helminths in communities treated [34], [35]. Addition of albendazole to ivermectin significantly reduced mf prevalence in mosquitos in the sentinel villages studied, which was an entomological confirmation of the importance of albendazole for LF control [36], [37]. This observation is related to our proposed hypothesis for the study (“areas previously exposed to ivermectin treatment for onchocerciasis control may require less rounds of annual MDA to eliminate LF”). Since the populations of the 12 districts had been exposed to ivermectin treatment for onchocerciasis control, this could have resulted in massive lowering of mf prevalence and density because ivermectin can reduce mf prevalence by up to 90%. The mf population was already under a selective pressure (based on the massive use of ivermectin), and this selective pressure was enhanced with the addition of a second drug (albendazole) to the MDA that has been occurring for years.

The successful implementation of the LF programme benefited from the existing onchocerciasis control programme by using CDTI as the platform [16]. The Onchocerciasis control programme was already well established using the CDTI strategy which allows communities to be in charge of all programme activities that are implemented within communities thus ensuring good sense of ownership and good compliance within communities. Health workers had already been trained and were available to provide technical support in additional training, supervision and surveys. Treatment has been given between September and December each year, as this is the period that was found to be convenient for the communities (i.e. harvest and post-harvest period). With integration of Onchocerciasis control and LF control CDTI plus (CDTI+) was adopted with the same principles as CDTI and Albendazole added to Ivermectin [16], [24], [35]. All the lessons learnt from CDTI during the years of the onchocerciasis control programme were used to improve the LF elimination programme, such as the use of the good health infrastructure in the districts that had focal persons for coordinating onchocerciasis control within districts to ensure a high treatment and geographic coverage by the national programme. These district onchocerciasis coordinators became district NTD coordinators. After the civil war in Sierra Leone in 2002, during which almost all health programmes had stopped, the MOHS had decided that running the onchocerciasis control programme as a vertical programmes would have been inefficient given the post war situation and the limited number of health workers and so the national coordination had to work in close collaboration with the DHMTs and within the existing district health structure. Furthermore, community directed interventions were continued for control of onchocerciasis and LF with which communities plan activities with health workers, decide treatment periods and select volunteers who are trained to distribute ivermectin and albendazole in their own communities.

Three rounds of MDA with compliance ≥65.0% in Papua New Guinea reduced mf prevalence from 18.6% to 1.3%, a 94.0% reduction [38]. The authors believed that the large decrease in prevalence occurred in part because the vector transmitting LF in the study area was the *Anopheles* mosquito, which is less efficient than *Culex* in the transmission of filariasis [38]. This may have also been the case in Sierra Leone. Similar successes in reducing mf prevalence after annual MDA rounds have been reported by many authors. In Kenya, there were similar reductions in mf prevalence (from 20.9% to 0.9%, a 95.7% reduction of mf prevalence) even when there were missed rounds of MDA [39]. Prevalence was reduced by 93.0%, from 12.0% to 0.8%, after just 2 rounds of MDA in Vanuatu [40]. In Northern Uganda, a reduction of mf prevalence from 3.7% to 0.4% (a 89.2% decrease) was reported after 3 MDAs [41]. Therefore, it is not surprising that three effective rounds of MDA would reduce the mf prevalence to below 1% in 11 out of 12 districts in the current LF elimination programme, given the relatively low mf prevalence at baseline.

The NTDCP in Sierra Leone has succeeded in building on an existing and effective CDTI programme for integrated management of onchocerciasis and LF and had the unique opportunity of using the integrated approach of managing both onchocerciasis and LF for LF elimination. As a result of the effectiveness of ivermectin alone in reducing LF in endemic communities baseline LF prevalence was relatively low. The NTDCP was able to use the good health infrastructure in the districts that had focal persons for coordinating NTD control within districts to ensure a high treatment and geographic coverage. Other countries embarking on LF elimination can learn the following lessons: in countries where the onchocerciasis control programme already exists and is successfully implemented, NTD control programmes can build on the existing CDTI structure for elimination of LF; integrated approach can be used for management of onchocerciasis and LF in areas co-endemic for onchocerciasis and LF (all activities can be co-implemented for the 2 diseases from training, community sensitization and mobilization to the MDA itself); in areas where CDTI has been implemented for many years programmes should expect to have relatively low baseline prevalence; integration of NTD control activities into strong existing national and district health system can ensure good programme implementation and improve programme sustainability especially for post MDA surveillance. Most African countries have problems providing adequate number of staff for public health programmes and integrating NTD programme into the national and district health system and co-implementation of activities for control of multiple NTDs can improve programme effectiveness and sustainability. It should be noted also that use of CDDs who do not get financial payments for the services they render may not work for MDAs in urban areas. The main difference noted in the NTDCP in Sierra Leone is that after the civil war 1991–2002, during which almost all health programmes had stopped, the MOHS had decided to put the control of all NTDs under the existing onchocerciasis control programme with 1(one) programme manager responsible for all NTDs and working in close collaboration with strong DHMTs. It was decided that running vertical programmes for NTDs will be disastrous given the post war situation and the limited number of health workers. This decision was easy to implement because before 2005–2008 when studies were conducted to map the other NTDs only the Onchocerciasis Control Programme was existing in the country.

The use of different sites for comparison (sentinel sites in 6 districts versus spot check sites for the other 6 districts) might be a limitation of the study considering the comparability of the impact assessment done in the districts where the same site was used relative to the districts where different sites were used. However, this depends on how you look at the study. In terms of the programme implementation it is not a limitation because recent WHO guidelines (WHO 2011) recommend 1 sentinel site per 1 million people. Only 1 district in Sierra Leone (the Urban Western Area, which is not in this group of 12 districts) has more than 1 million people and should have 1 sentinel site and 1 spot check site (total of 2 sites). The rest have far less than 1 million people per district and so have been grouped as recommended by WHO [22] depending on geographical proximity and epidemiological characteristics so that each pair has a total population of about 1 million people. Bonthe and Moyamba for example are geographically neighboring districts and have low baseline antigenemia and microfilaremia prevalence. The pair should have 1 sentinel site and 1 spot check site, so the sentinel site (selected and used for the baseline microfilaremia study) was used as sentinel site for the pair (in the case of Bonthe/Moyamba, Moboya in Bonthe was selected as a sentinel site) and a spot check site was selected in the other district as explained above (Taninahun Kapuima was recommended by the district health management team as good spot check site). The possible limitation for our paper is that we use the results obtained in the spot check sites and compare with baseline results in villages previously considered sentinel sites. Given the overall relatively low baseline microfilaremia prevalence and the pattern that emerges of a huge decrease noted in this mid-term evaluation, we believe that the impact assessment done in the districts where the same site was used relative to the districts where different sites were used are comparable if only to assess impact of MDA. In the case of Pujehun that had baseline mf prevalence of zero with the possibility that due to random selection the endemic areas (communities) might have been missed during the random selection of the sentinel sites at baseline, we think it is prudent to select and study another site/village that is indicated to be more LF endemic. In the pre-6^th^ MDA survey (pre-TAS), it will be recommended that another spot check site be selected, which is even more likely to be LF endemic in Pujehun to avoid risk of overlooking villages that could possibly still be a source of LF transmission within the district.

There is reason for optimism with the results of this survey because some research suggests that residual infections of filariasis disappear when prevalence is below 1.0% [42]. However, it is prudent to consider experiences and lessons learnt from other countries. In Tanzania, it was demonstrated that MDA using ivermectin and albendazole reduced mf prevalence by 21.2% and 40.4% after the first and second MDA respectively, but in subsequent MDAs, the effect leveled off and transmission, albeit low-level, was still noted after the third MDA [43]. In Leogane, Haiti, there was a significant reduction in mf rates after several rounds of MDA for LF, but transmission was not interrupted [44]. Mf prevalence detected after 3 MDAs does not demonstrate a change in filariasis transmission [38], [41]. The drug combination destroys the microfilaria over the 4–6 year it takes for the adult worm to die a natural death [38], [41], [45]. Therefore, MDA has to continue each year for 4–6 years, which is equivalent to the lifespan of the adult worm.

In conclusion, there was significant reduction of mf prevalence and density across the 12 rural districts in Sierra Leone after three annual MDAs. This was coupled with good MDA compliance and relatively low baseline endemicity. The results show that the proposed hypothesis is highly probable and that the LF elimination programme in Sierra Leone is on course to reach the objective of eliminating LF by the year 2020. Eliminating diseases such as LF has to follow models that use rigorous scientific data as is being demonstrated in this case. The next logical steps after the midterm evaluation include the following: continuation of annual MDAs for another 3 years (4^th^, 5^th^ and 6^th^ MDA rounds); a pre-transmission assessment survey (pre-TAS) before the 6^th^ MDA rounds; a TAS after the 6^th^ MDA rounds if district mf prevalence continue to be below 1%; and then 2 more TAS at intervals of 2–3 years before a request is made for certification of elimination. Manifestations of LF such as lymphoedema and hydroceles have to be included within the national surveillance system and monitored closely by the NTDCP.

## Supporting Information

Checklist S1 **STROBE Checklist.** Information/checkmarks were put against the sections of the STROBE checklist, used for reporting of observational studies, to indicate areas of the checklist covered in the manuscript.
(DOC)

## References

[1] Molyneux DH, Bradley M, Hoerauf A, Kyelem D, Taylor MJ (2003) Mass drug treatment for lymphatic filariasis and onchocerciasis. Trends Parasitol 19: 516–522. 14580963 10.1016/j.pt.2003.09.004

[2] WHO (2006) Preventive chemotherapy in human helminthiasis: coordinated use of anthelminthic drugs in control interventions. Geneva: World Health Organization.

[3] Ottesen EA, Hooper PJ, Bradley M, Biswas G (2008) The global programme to eliminate lymphatic filariasis: health impact after 8 years. PLoS Negl Trop Dis 2: e317. 18841205 10.1371/journal.pntd.0000317 PMC2556399

[4] WHO (2010) Global Programme to Eliminate Lymphatic Filariasis. Wkly Epidemiol Rec 85: 365–372. 20853547

[5] WHO (2011) Global Programme to Eliminate Lymphatic Filariasis: progress report on mass drug administration, 2010. Wkly Epidemiol Rec 86: 377–388. 21887884

[6] Boatin BA, Richards FO Jr (2006) Control of onchocerciasis. Adv Parasitol 61: 349–394. 16735169 10.1016/S0065-308X(05)61009-3

[7] WHO (2010) African Programme for Onchocerciasis Control - report of the sixth meeting of national task forces, October 2009. Wkly Epidemiol Rec 85: 23–28. 20095110

[8] Gbakima AA, Bockarie MJ, Sahr F, Palmer LT, Gooding E (2000) Rapid assessment of the prevalence and distribution of lymphatic filariasis in Sierra Leone. Ann Trop Med Parasitol 94: 299–301. 10884875 10.1080/00034980050006483

[9] Whitworth JA, Gilbert CE, Mabey DM, Morgan D, Foster A (1993) Visual loss in an onchocerciasis endemic community in Sierra Leone. Br J Ophthalmol 77: 30–32. 8435395 10.1136/bjo.77.1.30 PMC504418

[10] Mabey D, Whitworth JA, Eckstein M, Gilbert C, Maude G, et al (1996) The effects of multiple doses of ivermectin on ocular onchocerciasis. A six-year follow-up. Ophthalmology 103: 1001–1008. 8684787 10.1016/s0161-6420(96)30574-5

[11] Post RJ, Crosskey RW (1985) The distribution of the Simulium damnosum complex in Sierra Leone and its relation to onchocerciasis. Ann Trop Med Parasitol 79: 169–194. 4096566 10.1080/00034983.1985.11811905

[12] Dadzie KY, De Sole G, Remme J (1992) Ocular onchocerciasis and the intensity of infection in the community. IV. The degraded forest of Sierra Leone. Trop Med Parasitol 43: 75–79. 1519029

[13] Amazigo U (1999) Community selection of ivermectin distributors. Community Eye Health 12: 31. 17491996 PMC1706017

[14] The CDI Study Group (2010) Community-directed interventions for priority health problems in Africa: results of a multicountry study. Bull World Health Organ 88: 509–518 doi:10.2471/BLT.09.069203 20616970 10.2471/BLT.09.069203 PMC2897985

[15] Meredith SEO, Cross C, Amazigo UV (2012) Empowering communities in combating river blindness and the role of NGOs: case studies from Cameroon, Mali, Nigeria, and Uganda. Health Research Policy and Systems 10: 16. 22574885 10.1186/1478-4505-10-16 PMC3453498

[16] Hodges ME, Koroma JB, Sonnie M, Kennedy N, Cotter E, et al (2011) Neglected tropical disease control in post-war Sierra Leone using the Onchocerciasis Control Programme as a platform. International Health 3: 69–74. 24038179 10.1016/j.inhe.2011.03.003

[17] Hawking F (1957) The distribution of Bancroftian filariasis in Africa. Bull World Health Organ 16: 581–592. 13472412 PMC2538322

[18] Gbakima AA, Pessima J, Sahr F (1996) Parasitological and clinical studies on Wuchereria bamcrofti infectionin Moyamba District, Sierra Leone. Afr J Health Sci 3: 37–40. 17451295

[19] Brinkmann UK (1977) Epidemiological investigations of Bancroftian filariasis in the coastal zone Liberia. Tropenmed Parasitol 28: 71–76. 324055

[20] Zielke E, Chlebowsky HO (1979) Studies on bancroftian filariasis in Liberia, West Africa. II. Changes in microfilaraemia in a rural population some years after first examination. Tropenmed Parasitol 30: 153–156. 384627

[21] Koroma JB, Bangura MM, Hodges MH, Bah MS, Zhang Y, et al (2012) Lymphatic filariasis mapping by Immunochromatographic Test cards and baseline microfilaria survey prior to mass drug administration in Sierra Leone. Parasit Vectors 5: 10. 22236419 10.1186/1756-3305-5-10 PMC3268710

[22] WHO (2011) Monitoring and epidemiological assessment of mass drug administration: a manual for national elimination programmes. Geneva: World Health Organization.

[23] Hodges MH, Smith SJ, Fussum D, Koroma JB, Conteh A, et al (2010) High coverage of mass drug administration for lymphatic filariasis in rural and non-rural settings in the Western Area, Sierra Leone. Parasit Vectors 3: 120. 21162751 10.1186/1756-3305-3-120 PMC3018440

[24] WHO (2005) Monitoring and epidemiological assessment of the programme to eliminate lymphatic filariasis at the implementation unit level. Geneva: World Health Organization.

[25] Newcombe RG (1998) Two-sided confidence intervals for the single proportion: comparison of seven methods. Stat Med 17: 857–872. 9595616 10.1002/(sici)1097-0258(19980430)17:8<857::aid-sim777>3.0.co;2-e

[26] Koroma DS, Turay AB, Moihua MB (2006) Republic of Sierra Leone 2004 Population and Housing Census Statistics Sierra Leone. 25 p.

[27] Zoure HG, Wanji S, Noma M, Amazigo UV, Diggle PJ, et al (2011) The geographic distribution of Loa loa in Africa: results of large-scale implementation of the Rapid Assessment Procedure for Loiasis (RAPLOA). PLoS Negl Trop Dis 5: e1210. 21738809 10.1371/journal.pntd.0001210 PMC3125145

[28] El-Setouhy M, Abd Elaziz KM, Helmy H, Farid HA, Kamal HA, et al (2007) The effect of compliance on the impact of mass drug administration for elimination of lymphatic filariasis in Egypt. Am J Trop Med Hyg 77: 1069–1073. 18165524 PMC2196399

[29] Grady CA, de Rochars MB, Direny AN, Orelus JN, Wendt J, et al (2007) Endpoints for lymphatic filariasis programs. Emerg Infect Dis 13: 608–610. 17553278 10.3201/eid1304.061063 PMC2725965

[30] Huppatz C, Capuano C, Palmer K, Kelly PM, Durrheim DN (2009) Lessons from the Pacific programme to eliminate lymphatic filariasis: a case study of 5 countries. BMC Infect Dis 9: 92. 19523192 10.1186/1471-2334-9-92 PMC2702370

[31] Ottesen EA, Duke BO, Karam M, Behbehani K (1997) Strategies and tools for the control/elimination of lymphatic filariasis. Bull World Health Organ 75 6: 491–503. 9509621 PMC2487030

[32] Kyelem D, Sanou S, Boatin B, Medlock J, Coulibaly S, et al (2003) Impact of long-term ivermectin (Mectizan) on Wuchereria bancrofti and Mansonella perstans infections in Burkina Faso: strategic and policy implications. Ann Trop Med Parasitol 97 8: 827–838. 14754495 10.1179/000349803225002462

[33] Kyelem D, Medlock J, Sanou S, Bonkoungou M, Boatin B, et al (2005) Short communication: impact of long-term (14 years) bi-annual ivermectin treatment on Wuchereria bancrofti microfilaraemia. Trop Med Int Health 10 10: 1002–1004. 16185234 10.1111/j.1365-3156.2005.01489.x

[34] Ramaiah KD, Vanamail P, Pani SP, Yuvaraj J, Das PK (2002) The effect of six rounds of single dose mass treatment with diethylcarbamazine or ivermectin on *Wuchereria bancrofti* infection and its implications for lymphatic filariasis elimination. Tropical medicine and International health 7 9: 767–774. 12225508 10.1046/j.1365-3156.2002.00935.x

[35] Ramaiah KD, Das PK, Vanamail P, Pani SP (2003) The impact of six rounds of single dose mass administration of diethylcarbamazine or ivermectin on the transmission of *Wuchereria bancrofti* by *Culex quinquefasciatus* and its implications for lymphatic filariasis elimination programmes. Tropical medicine and International health 8 12: 1082–1092. 14641843 10.1046/j.1360-2276.2003.01138.x

[36] Richards FO, Pam DD, Kal A, Gerlong GY, Onyeka J, et al (2005) Significant decrease in the prevalence of *Wuchereria bancrofti* infection in anophelines mosquitoes following the addition of albendazole to annual, ivermectin-based, mass treatments in Nigeria. Annals of Trop. Medicine & Parasitology 99 2: 155–164. 10.1179/136485905X19838 15814034

[37] Richards FO, Eigege A, Pam D, Kal A, Lenhart A, et al (2005) Mass ivermectin treatment for onchocerciasis: Lack of evidence for collateral impact on transmission of *Wuchereria bancrofti* in areas of co-endemicity. Filaria Journal 4: 6 Http://www.filariajournal.com/content/4/1/6. 16022728 10.1186/1475-2883-4-6 PMC1208930

[38] Weil GJ, Kastens W, Susapu M, Laney SJ, Williams SA, et al (2008) The impact of repeated rounds of mass drug administration with diethylcarbamazine plus albendazole on bancroftian filariasis in Papua New Guinea. PLoS Negl Trop Dis 2: e344. 19065257 10.1371/journal.pntd.0000344 PMC2586652

[39] Njenga SM, Mwandawiro CS, Wamae CN, Mukoko DA, Omar AA, et al (2011) Sustained reduction in prevalence of lymphatic filariasis infection in spite of missed rounds of mass drug administration in an area under mosquito nets for malaria control. Parasit Vectors 4: 90. 21612649 10.1186/1756-3305-4-90 PMC3125382

[40] Fraser M, Taleo G, Taleo F, Yaviong J, Amos M, et al (2005) Evaluation of the program to eliminate lymphatic filariasis in Vanuatu following two years of mass drug administration implementation: results and methodologic approach. Am J Trop Med Hyg 73: 753–758. 16222021

[41] Ashton RA, Kyabayinze DJ, Opio T, Auma A, Edwards T, et al (2011) The impact of mass drug administration and long-lasting insecticidal net distribution on Wuchereria bancrofti infection in humans and mosquitoes: an observational study in northern Uganda. Parasit Vectors 4: 134. 21756371 10.1186/1756-3305-4-134 PMC3158553

[42] Mitja O, Paru R, Hays R, Griffin L, Laban N, et al (2011) The impact of a filariasis control program on Lihir Island, Papua New Guinea. PLoS Negl Trop Dis 5: e1286. 21886851 10.1371/journal.pntd.0001286 PMC3160343

[43] Simonsen PE, Pedersen EM, Rwegoshora RT, Malecela MN, Derua YA, et al (2010) Lymphatic filariasis control in Tanzania: effect of repeated mass drug administration with ivermectin and albendazole on infection and transmission. PLoS Negl Trop Dis 4: e696. 20532226 10.1371/journal.pntd.0000696 PMC2879369

[44] De Rochars MB, Kanjilal S, Direny AN, Radday J, Lafontant JG, et al (2005) The Leogane, Haiti demonstration project: decreased microfilaremia and program costs after three years of mass drug administration. Am J Trop Med Hyg 73 5: 888–894. 16282299

[45] Ottesen EA (2000) The global programme to eliminate lymphatic filariasis. Trop Med Int Health 5: 591–594. 11044272 10.1046/j.1365-3156.2000.00620.x

